# Quantification of tire tread wear particle in road dust through pyrolytic technique

**DOI:** 10.1016/j.heliyon.2023.e17796

**Published:** 2023-07-03

**Authors:** Eunji Chae, Sung-Seen Choi

**Affiliations:** Department of Chemistry, Sejong University, 209 Neungdong-ro, Gwangjin-gu, Seoul, 05006, Republic of Korea

**Keywords:** Tire wear particles (TWPs), Mineral particles (MPs), Quantification, Pyrolysis

## Abstract

Road dust cotains tire wear particles (TWPs) and a large amount of mineral particles (MPs). Given that tire tread in vehicles is mainly comprised of natural rubber (NR), isoprene and dipentene could be the main pyrogenic products stemmed from the thermolysis of NR. This offers a great chance to quantify the exact mass of TWP in road dust. As such, this study focused on the influence of MPs on the trends in thermolytic behaviors of NR using the resistive furnace (furnance) and Curie point pyrolyzers. This study confirmed that a reliable correlation in line with the formation of isoprene and dipentene could not be realized using the furnace type of a pyrolyzer. This means that employing the furnace type of a pyrolyzer in quantitification of TWPs could not be a viable and approproiate option due to the diverted thermolytic trends of NR due to differences in the heat transfer and adsoprtion of the pyrogenic products triggered by MPs. In the Curie point type of a pyrolyzer, the production rates of isoprene and dipentene were linearly responded to the mass of NR. The ferromagnetic substance in MPs could lead to the thermolytic trend change of NR. Thus, adopting the Curie point type of a pyrolyzer could be a viable option for quantification of TWPs in road dust when the effects of ferromagnetic substance are well neutralized.

## 1 Introduction

Road dust exhibits the heterogeneous nature, and this complexity has been mainly ascribed to an additional input of particles (generated from the traffic-related activities) and soil [[1], [2], [3], [4]]. As such, it is inferred that road dust contains a large amount of particulate matter (PM). In general, PM_10_ and PM_2.5_ are comprised of salts, crustal elements, organic materials, and so on [[5], [6], [7]]. Traffic-related particles also fall into the two categories (*i.e.,* exhaust and non-exhaust components) [8]. Non-exhaust components could be mostly comprised of particles stemmed from consecutive wearing of brakes, tires, and pavement [9,10]. Particularly, frictional contact between road and tire tread leads to abrasion of the tire tread. An abraded tire tread material without other particles is defined as tire wear particle (TWP), and TWP combined with other particles on the road is referred to tire-road wear particle (TRWP) [4,11]. As such, it is readily inferred that TRWPs are mainly composed of road and brake wear particles, soil, and so on [4]. In detail, the road wear materials are mixed up with the diverse minerals such as quartz, granite, quartzite, plagioclase, orthoclase, and ferromagnesian silicates [2,9,12].

Considering the global production of tires and other plastics, the fraction of microplastic mass attributable to tires could be appreciable and important for environmental risk management [13]. Tire wear emission in the whole world is estimated about 6 million tons per year [14]. In general, tire treads are made of natural rubber (NR), butadiene rubber (BR), and styrene-butadiene rubber (SBR) [[15], [16], [17]]. The tread compounds in the heavy duty vehicles (bus and truck) are mainly composed of NR [16,18]. Attenuated total reflectance-Fourier transform infrared spectroscopy (ATR-FTIR), nuclear magnetic resonance spectroscopy (NMR), and pyrolytic technique such as pyrolysis-gas chromatography/mass spectrometry (Py-GC/MS) have been used for analysis of raw and vulcanized rubbers [12,14,[19], [20], [21]]. Py-GC/MS has been practically used for identification and quantification of elastomers without sample pretreatment.

The three types of pyrolyzers (resistive furnace (furnace), Curie point, and microfurnace) are widely adopted in Py-GC/MS [[21], [22], [23], [24], [25]]. For the resistive furnace type of a pyrolyzer, a platinum filament coil or a ceramic heater is generally being used, and a quartz tube is used as a sample holder [21,24]. The Curie point type of a pyrolyzer is operated by wrapping a sample in a thin ferromagnetic foil (pyrofoil) with a specific Curie temperature [21,[23], [24], [25]]. A Curie point pyrolyzer instantly reaches up to its Curie temperature by losing its magnetic property [21,23,24]. Miller et al. analyzed cryomilled tire tread samples with or without a standard artificial sediment matrix, and reported that Curie point and microfurnace were good for quantitative analysis of TRWP using Py-GC/MS [23]. For the furnace type of a pyrolyzer, heat is transferred through the sample tube. If a sample is mixed with others, heat cannot transfer directly to the sample. Such fact causes the temperature deviation. However, for the Curie point type of a pyrolyzer, heat is directly transferred into the sample without heat transfer delay. Under the presence of the ferromagnetic materials mixed with the sample, the target temperature of the sample might be different from the Curie temperature of pyrofoil. Given that the field sample is a mixture of polymeric materials, mineral particles (MPs), and *etc*., it could be of great significance to count unexpected errors in quantitative analysis of TWPs in road dust.

A considerable amount of road dust could be generated by friction between tire tread and road nearby bus stops. To determine suitability and error level, this study focused on the thermolytic behaviors of TWPs by means of a series of the pyrolysis tests of NR using the two types of a pyrolyzer. In specific, a mixture of NR and MPs was pyrolyzed using the two types of a pyrolyzer, and the formation of isoprene and dipentene varied with a mass of NR and a size of MPs were monitored. Note that granite powder was used as a model MP in this study. The reference sample of NR was intentionally pulverized to simulate real TWP. Road dust was collected at a bus stop and an existence of TWPs in road dust was scrutinized.

## 2 Materials and methods

### 2.1 Materials and preparation of model MPs and road dust samples

SMR CV 60 was employed as NR and toluene was purchaed from J. T. Baker (USA). Model MPs were prepared by breaking granitic rock (Pocheonseok supplied from Dongastone Co., Republic of Korea) into powder with a hammer and by following separation by size using a sieve shaker of Octagon 200 (Endecotts Co., UK). Standard sieves with aperture of 1000, 500, 212, 106, 63, 38 μm (Endecotts Co., UK) were used. Size separation was performed with the interval mode for 20 min. MPs of 212–500, 106–212, 63–106, and 38–63 μm were used. Shapes of the MPs were observed using an image analyzer (EGVM35B, EG Tech., Republic of Korea) (Figure S1; Supplementary Information).

Road dust was collected at a bus stop (37°33′12.6"N 127°04′23.5"E). The road dust was separated by size using a sieve shaker. The road dust samples of 212–500, 106–212, 63–106, and 38–63 μm were used for analysis.

### 2.2 Preparation of NR particle samples

The initial NR solution of 10.0 mg mL^−1^ was prepared by dissolving SMR CV 60 in toluene. This NR solution was diluted to 2.0 and 0.40 mg mL^−1^. An NR particle sample was prepared as follows: (1) the NR solution was dropped onto a slide glass, (2) the solvent was evaporated in a convection oven at 70 °C for 30 min, and (3) a solid NR particle was obtained by gathering the dried NR with a knife. The NR particle samples of 10.0 and 20.0 μg were obatined using the 2.0 mg/mL NR solution of 5.0 and 10.0 μL, respectively, while those of 2.0 and 5.0 μg were obatined using the 0.40 mg mL^−1^ NR solution of 5.0 and 12.5 μL, respectively.

### 2.3 Sample preparation and pyrolysis conditions for the furnace type of a pyrolyzer

**Fig. 1:**
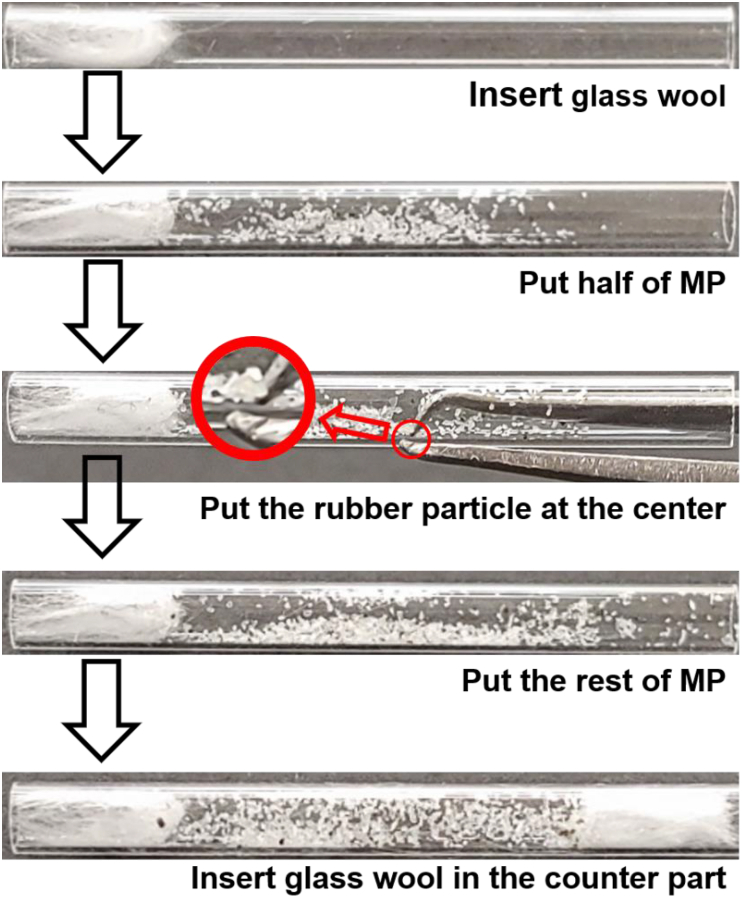
Sample preparation procedure of mixture of NR and MPs for furnace type pyrolysis.

A furnace type pyrolyzer of a pyroprobe 2000 system with a CDS 1500 interface (Chemical Data System, Oxford, USA) was used. The furnace type pyrolyzer uses a quartz tube for the sample holder. Weight of the MPs was 5.5 mg. The sample preparation process for furnace type pyrolysis was as follows (Fig. 1): (1) insert glass wool into the one end part of the quartz tube, (2) put half of the MPs into the quartz tube, (3) put the rubber particle at the center of the quartz tube, (4) put the rest of the MPs into the quartz tube, and (5) insert glass wool into the other end part of the quartz tube. The sample was pyrolyzed at 590 °C for 10 s under helium (He) atmosphere.

### 2.4 Sample preparation and pyrolysis conditions for the Curie point type of a pyrolyzer

**Fig. 2:**
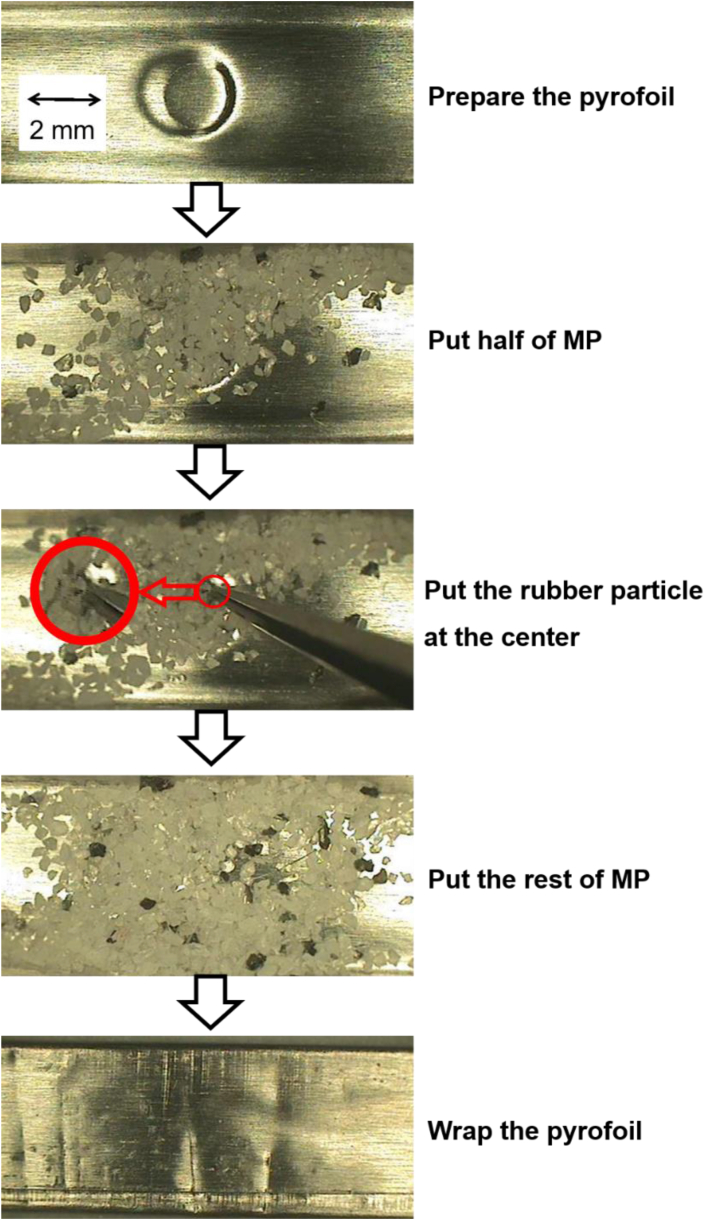
Sample preparation procedure of mixture of NR and MPs for Curie point pyrolysis.

JCI-55 Ci-point pyrolyzer (Japan Analytical Industry Co., Japan) was used. The Curie point pyrolyzer uses a pyrofoil for analysis. Weight of the MPs was 5.5 mg. The sample preparation process for Curie point pyrolysis was as follows (Fig. 2): (1) make the pyrofoil U-shaped for sample loading, (2) put half of the MPs on the pyrofoil, (3) put the rubber particle on the MPs, (4) put the rest of the MPs on the rubber particle, and (5) wrap the pyrofoil. A pyrofoil of 590 °C Curie temperature was used. Pyrolysis was performed for 10 s under an inert gas (He) environment.

### 2.5 Analysis conditions of GC/MS

An Agilent 6890 GC (Agilent Technology Inc., USA) equipped with an Agilent 5973 MSD (Agilent Technology Inc., USA) was used. DB-5MS (30 m × 0.32 mm, film thickness 0.25 μm, Agilent Technology Inc., USA) was used. The sample inlet temperature was 250 °C, the split ratio was 1:15, and helium (1.8 mL⋅min^−1^) was used as the carrier gas. The GC oven temperature programming was as follows: 30 °C (held for 3 min) to 160 °C at a rate of 8 °C⋅min^−1^ (held for 1 min), and then to 250 °C at a rate of 10 °C⋅min^−1^ (held for 3 min). The interface temperature of GC to MS was 250 °C. The electron ionization (70 eV) was used to ionize the pyrolysis products. The MS source temperature was 230 °C.

## 3 Results and discussion

### 3.1 Influence of MPs on the thermolytic behavior of NR by furnace type pyrolysis

Py-GC/MS chromatograms of the NR/MP mixture samples pyrolyzed using the furnace type of a pyrolyzer show isoprene and dipentene peaks at 1.35 and 10.17 min, respectively (Figures S2(a) and (b), Supplementary Information). Isoprene and dipentene are the principal pyrogenic products of the thermolysis of NR (Schemes S1 and S2, Supplementary Information). The peak intensities of isoprene and dipentene were different depending on the size of MPs, and there was no specific trends. For the sample of 5.0 μg NR, the intensities of isoprene and dipentene were the greatest when mixing with the MPs of 38–63 μm whereas dipentene was not observed when mixing with the MPs of 63–106 μm. Py-GC/MS chromatograms of the samples of 20.0 μg NR were very different from those of the samples of 5.0 μg NR. For the sample of 20.0 μg NR, the abundances of isoprene and dipentene were much lower when mixing with the MPs of 38–63 and 63–106 μm than those when mixing with the MPs of 106–212 and 212–500 μm.

**Fig. 3:**
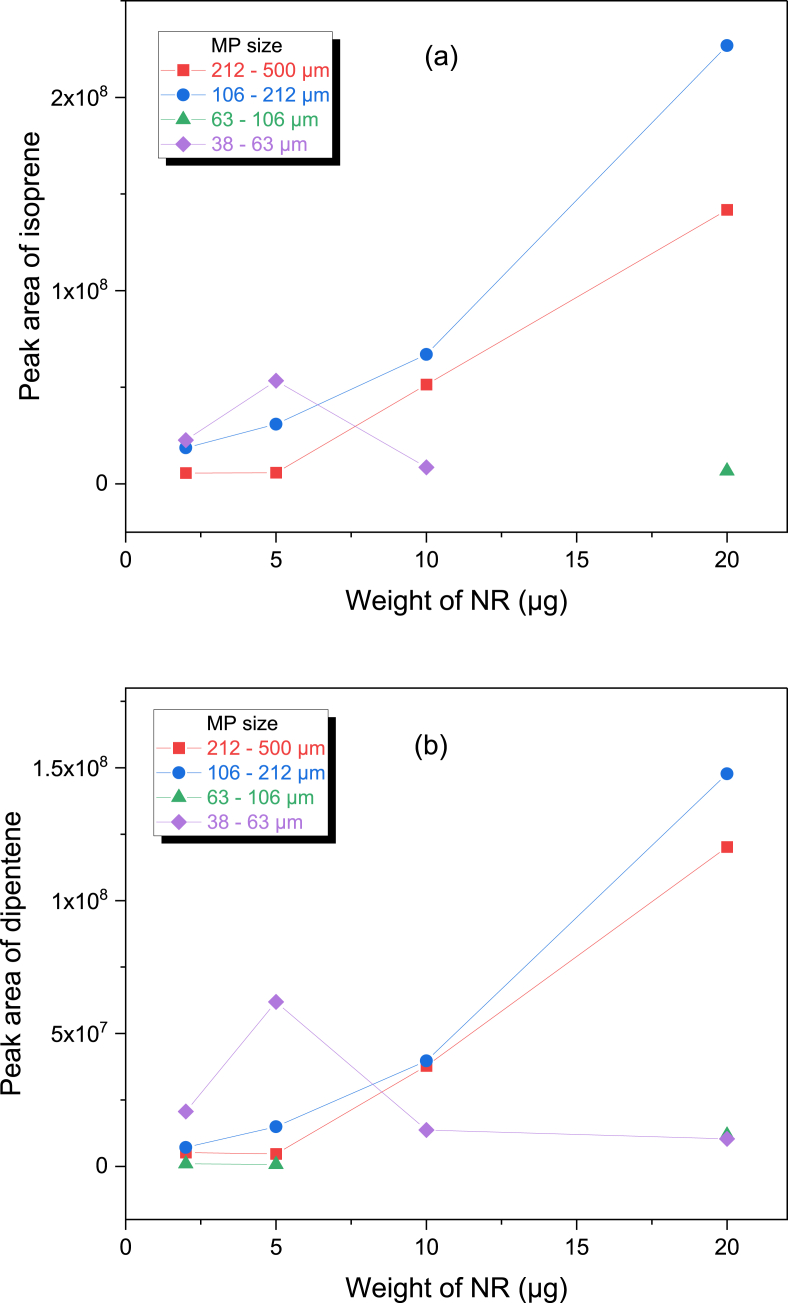
Variations of the peak areas of isoprene (a) and dipentene (b) produced from the mixture of NR and MPs by the furnace type pyrolysis with the NR weight.

The intensities of isoprene and dipentene were plotted with varied mass of NR and size of MPs to seek the intensity differences (Fig. 3(a) and (b), respectively). For the MPs of 106–212 and 212–500 μm, the intensity of isoprene tended to increase as the mass of NR increased. But the isoprene was not observed for the sample of 20.0 μg NR and 38–63 μm MP, and it was detected only by a trace for the sample of 20.0 μg NR and 63–106 μm MP. The dipentene intensities of the samples of 106–212 and 212–500 μm MP tended to increase as the mass of NR increased. The dipentene intensity of the sample of 5.0 μg NR and 38–63 μm MP was much greater than those of the 10.0 and 20.0 μg NR samples. In summary, when the NR/MP mixture samples were pyrolyzed using the furnace type of a pyrolyzer, the abundances of isoprene and dipentene were different from each other. This can be explained by the differences in heat transfer and adsorption of some pyrogenic products on MPs.

For the furnace type of a pyrolyzer, the particle of NR in the sample tube was heated through the quartz tube. As such, the particle of NR will be indirectly heated through MPs if it is surrounded by MPs. Thermal conductivity of granite is 1.2–1.3 W m^−1^ K^−1^ at 873 K, which is greater than that of helium (0.3 ⋅m^−1^⋅K^−1^ at 800 K) [26,27]. Heat transfer conditions should be changed in the presence of MPs, and the real tempearture targetted the NR particle could be very different from each other. The pyrolysis temperature is a key operational parameter for govering the kinds and abundances of the pyrogenic products [28]. Another influencing factor was adsorption of some pyrogenic products on MPs. The MPs changed from light to dark colors (Figure S3, Supplementary Information). This is likely due to adsorption of some pyrogenic products on MPs. By decreasing the size of MPs, color of the MPs was getting darker after the pyrolysis test. Thus, quantitfication of NR mixed with MPs using the furnace type of a pyrolyzer has unavoidable errors. It was also concluded that the furnace type of a pyrolyzer could not be appropriate in quantitative analysis of NR mixed with MPs.

### 3.2 Analysis of road dust using furnace type pyrolysis

**Fig. 4:**
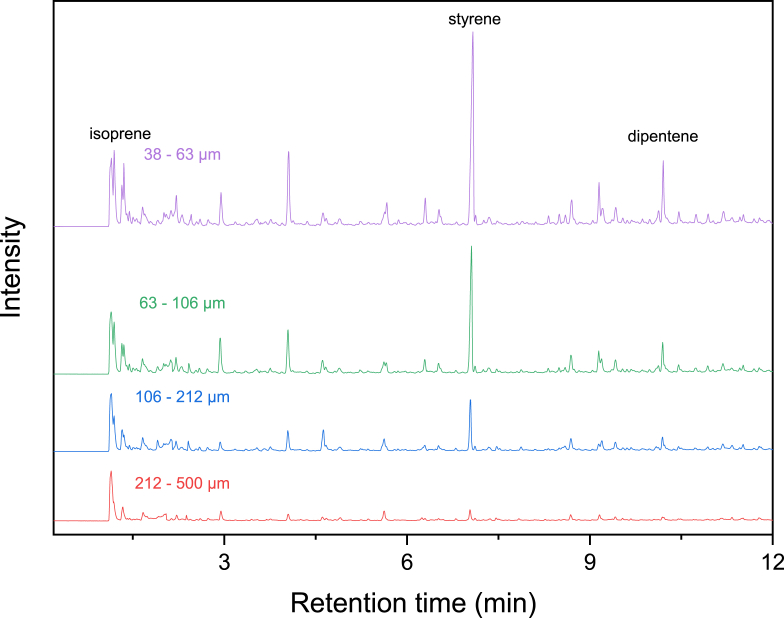
Py-GC/MS chromatograms of the road dust samples pyrolyzed by furnace type pyrolysis.

**Fig. 5:**
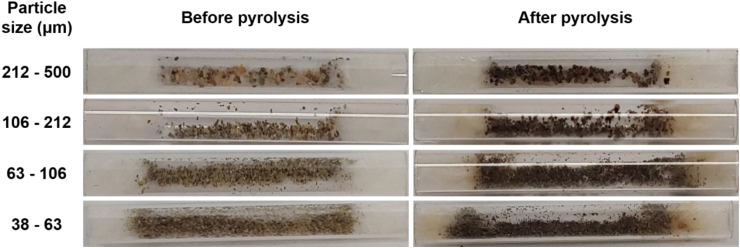
Photos of the road dust samples in the sample tube before and after the furnace type pyrolysis.

Fig. 4 shows Py-GC/MS chromatograms developed from road dust using the furnace type of a pyrolyzer. Road dust contains TRWPs, asphalt pavement wear particles (APWPs), MPs, and *etc.* [29]. As claimed earlier, there must be NR in the road dust because tire tread for heavy-duty vehicles (such as a bus) is mainly comprised of NR. In reference to the Py-GC/MS chromatograms of the NR samples mixed with the MPs (Figure S2, Supplementary Information), the more pyrogenic products were detected. Isoprene and dipentene were detected in the chromatograms. One of the most abundant pyrogenic products was styrene which may be stemmed from bitumen in asphalt pavement. The peak areas of isoprene and dipentene increased by decreasing the size of road dust (Table S1, Supplementary Information). This infers that the content of TWP in road dust with the smaller size was higher than that with the larger size. 4-Vinylcyclohexene (VCH) was also detected in the road dust samples of 38–63 and 63–106 μm. It is a key index pyrogenic product stemmed from the thermolyses of BR and SBR. Hence, detection of VCH infers the presence of BR or SBR in the road dust. Fig. 5 shows the quartz tubes before and after the pyrolysis of the road dust samples. The darkness got deeper after pyrolysis and the glasswool was also turned brown. This color change of glasswool tended to be darker by decreasing the size of road dust. The color changes of the road dust after pyrolysis implies that there were various organic matter in road dust.

### 3.3 Influence of MPs on the thermolytic behavior of NR by Curie point pyrolysis

**Fig. 6:**
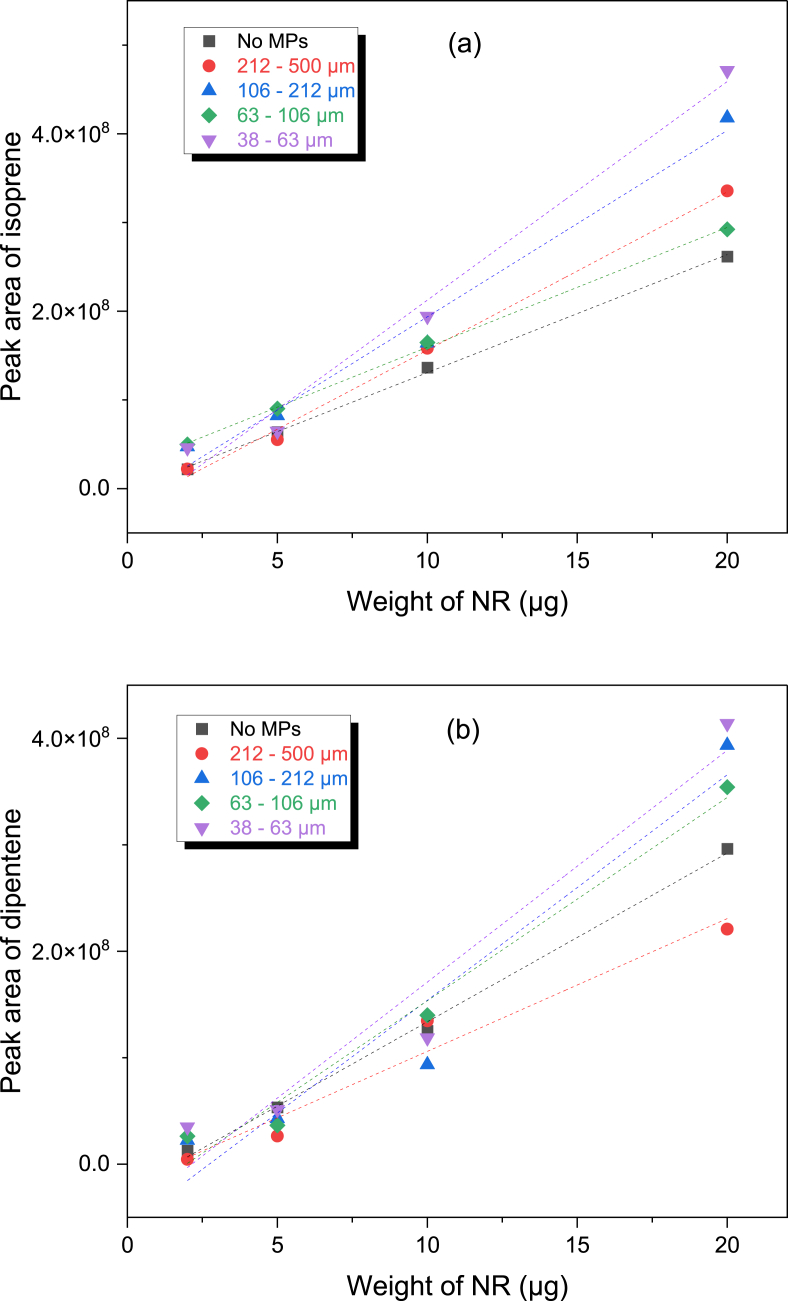
Variations of the peak areas of isoprene (a) and dipentene (b) produced from the mixture of NR and MPs by the Curie point pyrolysis with the NR weight.

A Py-GC/MS chromatogram of the mixture samples developed from the Curie point type of a pyrolyzer shows the real presence of isoprene and dipentene (Figures S4(a) and (b), Supplementary Information). Intensity variations of isoprene and dipentene were plotted as a function of the NR mass as presented in Fig. 6(a) and (b), respectively. The abundances of isoprene and dipentene of pure NR without MPs as the reference were also plotted. Except for the NR 5.0 μg mixed with the MPs of 212–500 and 38–63 μm, the isoprene intensities of the mixture samples were larger than those of the pure one. This may be due to the ferromagnetic materials in MPs. The ferromagnetic substance can release heat by an applied field. Hence, the real temperature applied to the particle of NR should be higher than the Curie temperature of the pyrofoil due to the ferromagnetic substance in MPs. The isoprene intensity increased linearly by increasing the NR mass, irrespective of the size of MPs. The isoprene abundances of the mixture samples of 2.0 μg NR were greater than that of the pure NR sample by over twice. By decreasing the size of MP, the abundance of isoprene tended to slightly increase.

The intensities of dipentene also tended to increase as the NR mass increased, irrespective of the size of MPs as shown in Fig. 6(b). Some mixture samples exhibited the stronger inteneities of dipentene than the pure NR samples, but others did not. For the mixture samples of 2.0 and 20.0 μg NR, the abundances of dipentene were higher than the pure NR sample except for the samples of 212–500 μm MP. The dipentene abundances of the mixture samples of 5.0 μg NR were lower than that of the pure NR sample.

Indeed, there are three major factors affecting the thermolytic behavior of NR by Curie point pyrolysis of the NR/MP mixture. First, blocking of heat transfer from pyrofoil to NR by MPs makes the temperature lower. Second, the ferromagnetic substance in MPs makes the temperature higher. Lastly, adsorption of some pyrogenic products on MPs reduces detection of the pyrogenic products. Among them, the most critical factor could be the ferromagnetic substance because the NR/MP mixture produced isoprene greater than the pure NR. Granite powder used in this study as the model MPs consists of varios metal oxides such as silicon oxide (SiO_2_), aluminum oxide (Al_2_O_3_), and iron oxides (FeO and Fe_2_O_3_), and metal elements such as Fe, Ba, Ni, and Cu [30]. Among them, Fe, Ni, and Fe_2_O_3_ are ferromagnetic, and their Curie temperatures are 770, 358, and 680 °C, respectively [31,32]. As the thermolytic temperature increases, the production rate of a monomer is greater than that of a dimer. If the particle of NR is surrounded by MPs with the ferromagnetic properties, the pyrolysis temperature will rise locally and isoprene may be more produced.

Colors and shapes of the MPs in pyrofoils before and after the pyrolysis test of the mixture samples were examined (Figure S5, Supplementary Information). Some MPs were more broken into smaller pieces by folding pyrofoil. Some of MPs changed to brown color after the pyrolysis, but the degree of color change was less than the furnace type pyrolysis. This implies that some pyrogenic products were adsorbed on MPs but the degree of adsoprtion could be lower than the case of the furnace type pyrolysis. Plots for the peak areas of isoprene and dipentene produced by the Curie point pyrolysis with the NR weight in Fig. 6 were curve-fitted. The linearity for the curve-fitted equations was good; the correlation coefficients were >0.990 and > 0.960 for the plots of isoprene and dipentene, respectively. The average slopes for the plots of isoprene excluding and including data for the pure NR sample were 1.92(±0.57) × 10^7^ and 1.80(±0.66) × 10^7^, respectively, while those of dipentene were 1.84(±0.59) × 10^7^ and 1.81(±0.56) × 10^7^, respectively. Though quantification of NR mixed with MPs using the Curie point type of a pyrolyzer has some errors, the tolerances could be acceptable. Thus, it was also concluded that adopting the Curie point type of a pyrolyzer could be a viable option for quantitative analysis of NR mixed with MPs.

### 3.4 Analysis of road dust using Curie point pyrolysis

**Fig. 7:**
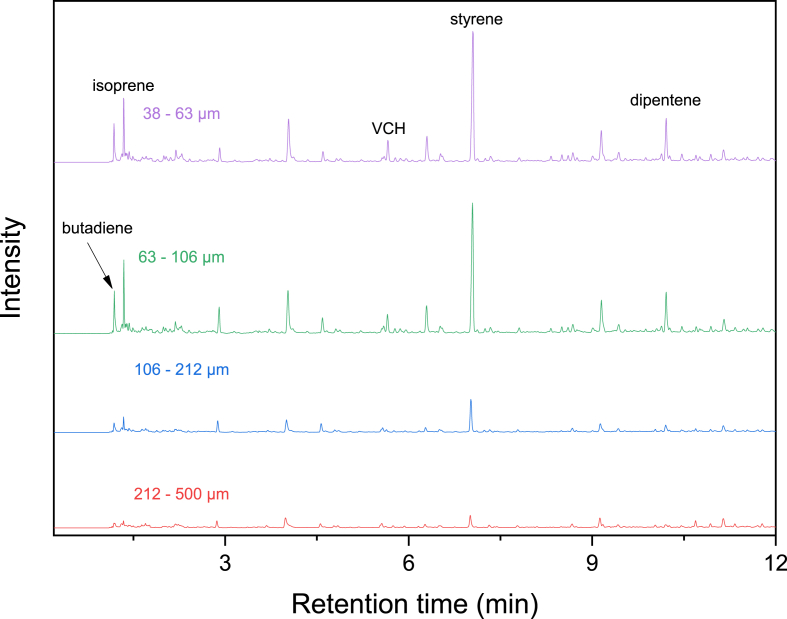
Py-GC/MS chromatograms of the road dust samples pyrolyzed by the Curie point pyrolyzer.

**Fig. 8:**
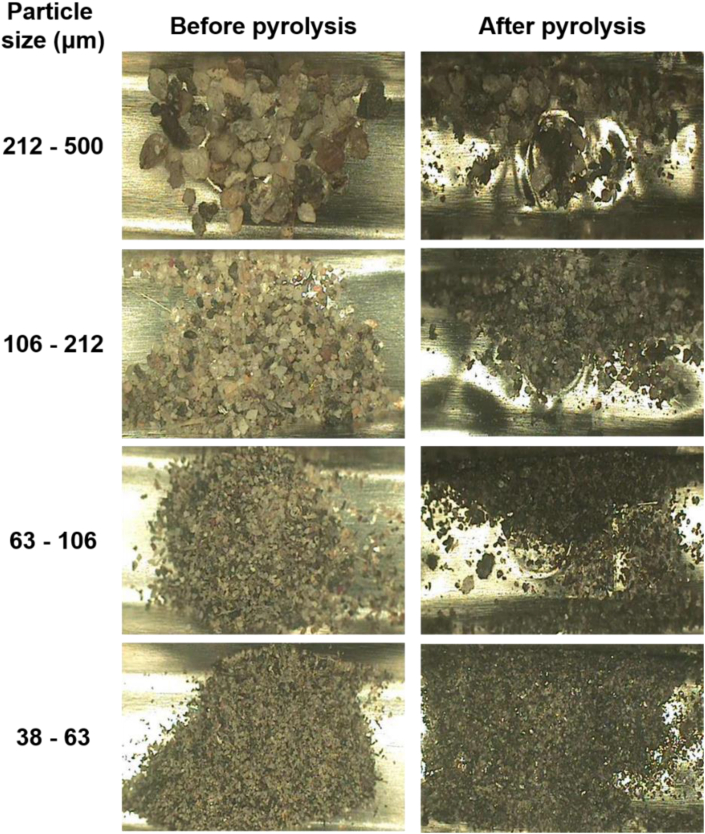
Photos ( × 40) of the road dust samples on the pyrofoil before and after the Curie point pyrolysis.

Fig. 7 shows Py-GC/MS chromatograms of the road dust samples using the Curie point type of a pyrolyzer. Lots of the pyrogenic products were identified. Besides isoprene, dipentene, and styrene, VCH was also observed. VCH was detected in the road dust samples of 38–63 and 63–106 μm like the analysis results developed from the furnace type of a pyrolyzer (Fig. 4). Since VCH is the principal pyrolysis product of BR and SBR, detection of VCH indicates that the road dust contains BR or SBR. The intensities of isoprene and dipentene increased as the size of road dust decreased (Table S2, Supplementary Information). This means that the content of TWP related to NR in the road dust with the smaller size was higher than that in the road dust with the larger size. Fig. 8 shows the pyrofoils containing road dust before and after the pyrolysis test. Color of the particles was getting darker after the pyrolysis. Color of the road dust changed more than that of the NR/MP mixture samples. This is because the road dust contained bitumen (APWPs) and other organic components as well as TRWPs.

### 3.5 Influence of reference NR states on the thermolytic behavior of NR

According to ISO/TS 20593 (Ambient air - Determination of the mass concentration of tire and road wear particles (TRWP) - Pyrolysis-GC/MS method), deuterated polyisoprene solution was used as the internal standard for quantification of NR in PM sample and chloroform was used as the solvent. 5.0 mg of NR was dissolved in 10.0 mL chloroform to prepare 0.50 mg NR mL^−1^ solution. Two kinds of NR/MP samples were prepared using the 10.0 μL NR solution and 5.0 μg NR particle made of the 10.0 μL NR solution, respectively. According to the ISO/TS 20593, the internal standard solution was dropped on an environmental sample. Like the same way, the NR solution of 10.0 μL was dropped on the MP of 5.5 mg. The sample using the particle of NR was prepared as mentioned in section 2, 3, 4. The analysis results were summarized in Table 1. The intensities of isoprene and dipentene for the NR solution were greater than those for the NR particle. This indicates that use of the NR solution has better pyrolysis efficiency than use of the NR particle, but this also denotes that use of the NR solution can underestimate the NR content in the sample. The error ranges for the NR solution were larger than those for the particle of NR. This implies that use of the NR solution can cause larger error than use of the particle of NR as the internal standard.

**Table 1:** Peak areas of isoprene and dipentene in the Py-GC/MS chromatograms of the reference NR samples depending on the sample states (solution and particle). Chloroform was used as the solvent and Curie point pyrolyzer was used. Weights of the NR and MP were 5.0 μg and 5.5 mg, respectively. The same sample was analyzed five times and averaged.

	Isoprene	Dipentene
Solution	2.90 × 10^7^±0.52 × 10^7^ (±17.9%)	2.52 × 10^7^±0.46 × 10^7^ (±18.3%)
Particle	2.70 × 10^7^±0.28 × 10^7^ (±10.4%)	2.04 × 10^7^±0.33 × 10^7^ (±16.2%)

### 3.6 Considering factors for quantitative analysis of TWP content in environmental samples

As claimed, adopting the Curie point type of a pyrolyzer is could be better for quantification of TWPs. Considering factors for quantitative analysis of TWPs in road dust and sediment were as follows: (1) the ferromagnetic substance, (2) indirect heat transfer, (3) adsorption of the pyrogenic products, (4) the type of an internal standard. The ferromagnetic substance in a sample leads to increase the temperature. There are lots of mineral particles in the environmental samples and some of them have the ferromagnetic property. It is not easy to exactly determine the amount of the ferromagnetic substance in a sample. Thus, errors occurred by the ferromagnetic substance are not avoidable. Indirect heat transfer by MPs can lower the real pyrolysis temeprature to reduce the amounts of the pyrogenic products. Adsorption of the pyrogenic products by MPs leads to lower detection efficiency of the pyrogenic products. The thermolytic behavior of the internal standard is also affected by MPs and the error level could not be negligible. The internal standard with particle type is closer to real TWPs than the solution type and the former showed smaller error range than the latter.

## 4 Conclusion

The abundances of isoprene and dipentene produced from the NR/MP mixture samples by the furnace type pyrolysis were very different from each other. Factors influencing the thermolytic behavior of NR in the mixture samples for the furnace type of a pyrolyzer were the differences in heat transfer and adsorption of some pyrogenic products by MPs. Since quantification of NR mixed with MPs using the furnace type of a pyrolyzer was not reliable, this method was not proper for quantification of TWPs. For the Curie point type of a pyrolyzer, the intensities of isoprene and dipentene linearly increased by increasing the NR mass irrespective of the size of MPs. Factors influencing the thermolytic behavior of NR in the mixture samples for Curie point pyrolysis were blocking of direct heat transfer from pyrofoil to NR, the ferromagnetic substance in MPs, and adsorption of some pyrogenic products on MPs. The Curie point type of a pyrolyzer was relatively reliable for quantification of TWP in road dust and sediment. Considering factors for the quantification of TWPs in environmental samples are the type of pyrolyzer, inhomogeneity of sample, blocking of direct heat transfer, the ferromagnetic substance, and adsorption of pyrogenic products. It was also concluded that the internal standard of particle type was relatively more reliable than that of solution type.

## Author contribution statement

Eunji Chae: Conceived and designed the experiments; Performed the experiments; Analyzed and interpreted the data; Wrote the paper.

Sung-Seen Choi: Conceived and designed the experiments; Contributed reagents, materials, analysis tools or data; Wrote the paper.

## Data availability statement

Data will be made available on request.

## Additional information

Supplementary content related to this article has been published online at [URL].

## Funding

This work was supported by the Technology Innovation Program funded by the 10.13039/501100003052Ministry of Trade, Industry and Energy, Republic of Korea (Project Number 20010851), and this research was supported by R&D Program funded the 10.13039/501100003052Ministry of Trade, Industry and Energy, Republic of Korea (Project Number 20003587).

## Declaration of competing interest

The authors declare that they have no known competing financial interests or personal relationships that could have appeared to influence the work reported in this paper.
