# Supplementary material for: Quantification of tire tread wear particle in road dust through pyrolytic technique
Source: Heliyon. 2023 Jul 3;9(7):e17796. doi: 10.1016/j.heliyon.2023.e17796 (PMC10359866; doi:10.1016/j.heliyon.2023.e17796)
Supplement: Multimedia component 1 [file mmc1.docx]

**Quantification of tire tread wear particle in road dust through pyrolytic technique**

**Supplementary information**

**Table S1**

Peak areas of isoprene and dipentene in the Py-GC/MS chromatograms of the road dust samples. Furnace type pyrolyzer was used.

| Particle size of  the road dust (μm) | Peak area (10^6^) | |
| --- | --- | --- |
|  | Isoprene | Dipentene |
| 38 - 63 | 77.9 | 156.4 |
| 63 - 106 | 31.5 | 70.9 |
| 106 - 212 | 9.4 | 27.5 |
| 212 - 500 | 9.4 | 27.6 |

**Table S2**

Peak areas of isoprene and dipentene in the Py-GC/MS chromatograms of the road dust samples. Curie point pyrolyzer was used.

| Particle size of  the road dust (μm) | Peak area (10^6^) | |
| --- | --- | --- |
|  | Isoprene | Dipentene |
| 38 - 63 | 73.4 | 95.0 |
| 63 - 106 | 70.0 | 91.0 |
| 106 - 212 | 14.0 | 16.4 |
| 212 - 500 | 4.7 | 5.5 |


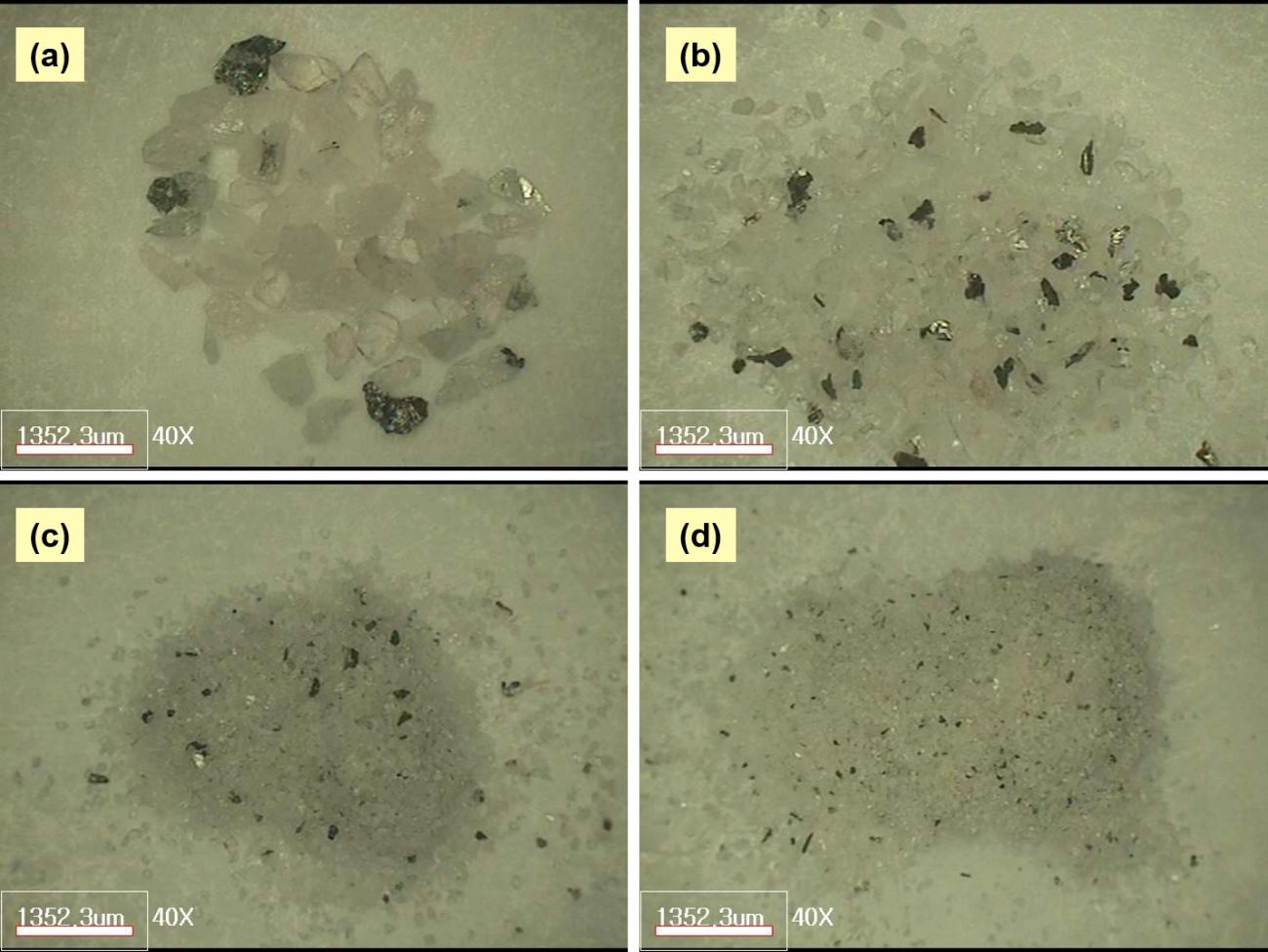


**Fig. S1.** Magnified images (× 40) of 5.5 mg mineral particles of (a) 212 - 500, (b) 106 – 212, (c) 63 – 106, and (d) 38 - 63 μm.

**Fig. S2.** Py-GC/MS chromatograms of the mixture of NR and MPs pyrolyzed by the furnace type pyrolyzer. The MP weight is 5.5 mg, and the NR sample weights are (a) 5.0 and (b) 20.0 μg.


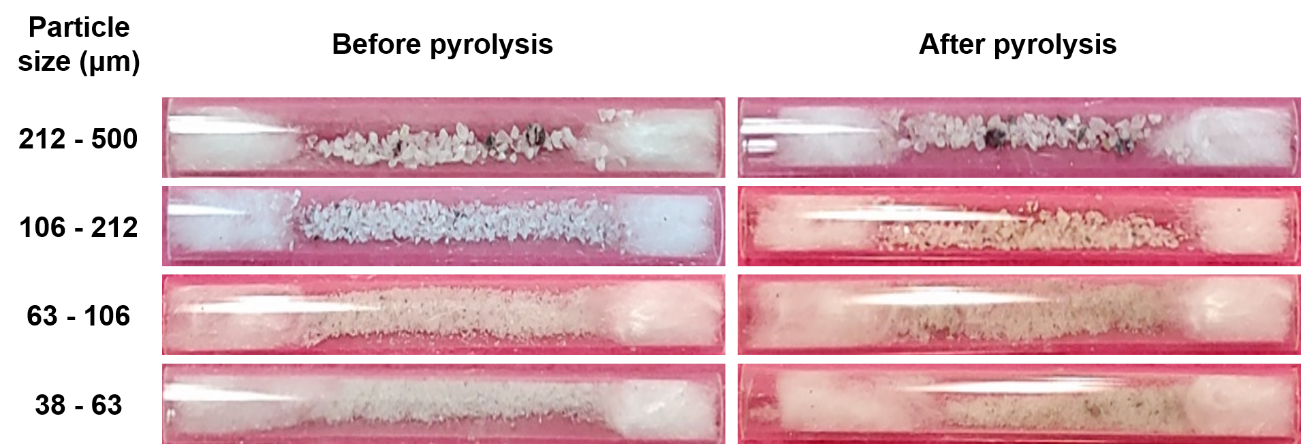


**Fig. S3.** Photos of the mixture of 10 μg NR and MPs in the sample tube before and after the furnace type pyrolysis.

**Fig. S4.** Py-GC/MS chromatograms of the mixture of NR and MPs pyrolyzed by the Curie point pyrolyzer. The MP weight is 5.5 mg, and the NR sample weights are (a) 5.0 and (b) 20.0 μg.


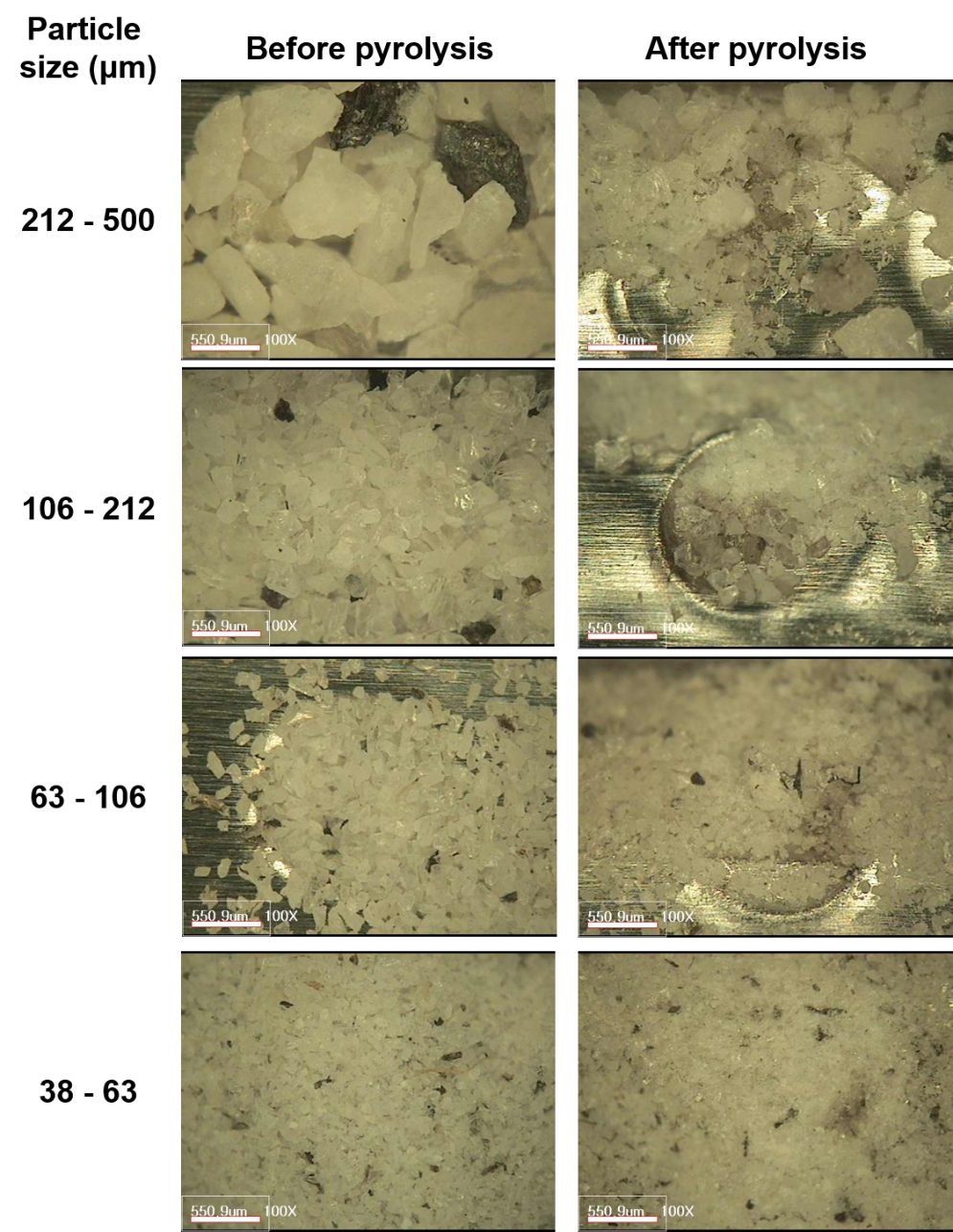


**Fig. S5.** Photos (× 100) of the mixture of 10 μg NR and MPs on the pyrofoil before and after the Curie point pyrolysis.

**Scheme S1.** Pyrolysis mechanism for formation of isoprene from NR.

**Scheme S2.** Pyrolysis mechanism for formation of dipentene from NR.
